# Diagnostic Values of Dermatoscopy and CD31 Expression in Cutaneous Lymphangioma Circumscriptum

**DOI:** 10.3389/fmed.2021.738815

**Published:** 2021-10-08

**Authors:** Lixia Lu, Siyu Yan, Mingliang Chen, Xiaoyan Huang, Juan Su

**Affiliations:** ^1^Department of Dermatology, Xiangya Hospital, Central South University, Changsha, China; ^2^Hunan Key Laboratory of Skin Cancer and Psoriasis, Central South University, Changsha, China; ^3^Hunan Engineering Research Center of Skin Health and Disease, Central South University, Changsha, China; ^4^Department of Dermatology, The Third Xiangya Hospital, Central South University, Changsha, China; ^5^Department of Pathology, Xiangya Hospital, Central South University, Changsha, China

**Keywords:** dermoscopy, immunohistochemistry, CD31, dermatoscope, cutaneous lymphangioma circumscriptum

## Abstract

**Background:** Cutaneous lymphangioma circumscriptum is characterized by clusters of deep-seated, vesicle-like papules. Cutaneous lymphangioma circumscriptum (CLC) is not a tumor but rather a congenital malformation of superficial lymphatics.

**Objectives:** The study aimed to describe the dermoscopic features of CLC and investigate the reason why marked blood components in CLC. Moreover, this study sought to increase awareness of the clinical characteristics of CLC and provide insights into CLC diagnosis.

**Methods:** A representative sample of patients with CLC with demographic information and pathological and dermoscopical results was analyzed. The immunohistochemistry of lymphangioma specimens with CD31 and D2-40 was performed. The clinical manifestations of CLC, demographic information, and the results of immunohistochemistry were statistically analyzed to validate the correlation.

**Results:** Besides the pattern of frog spawn-like blisters, lymphangioma also presented as either transparent or pigmented with dark-red to whitish/yellowish shades. Moreover, lymphangioma manifested as a pattern of dermatofibroma. Furthermore, CD31 was detected in the flattened endothelium and only present in dilated spaces containing enough blood or lymph components.

**Limitations:** This study is limited by its retrospective nature and statistical power.

**Conclusion:** Dermoscopy is useful for the diagnosis of CLC. CD31 positive staining and cystic-dilated spaces showed flattened inner and outer endothelia are the diagnostic features in hypopyon-like shape and blisters resembling frog spawn patterns in CLC. These features can assist in the diagnosis of CLC.

## Introduction

Cutaneous lymphangioma circumscriptum is a benign disorder with asymptomatic lesions, including discrete translucent vesicles (1, 2). Some of the common characteristics of cutaneous lymphangioma circumscriptum (CLC) are the presence of a group of transparent vesicles that are 2–4 mm in size, patterns similar to frog spawn, and diversity in color as a result of hemoglobin degradation (3, 4). These skin lesions are located in any part of the body, and their color varies from clear to dark red depending on the presence of lymphatic fluid and/or blood components (3). CLC manifests striking characteristic features, differentiating it from other lymphatic or vascular diseases such as hemangiomas (5).

Dermoscopy is a non-invasive clinical diagnostic technique wherein the skin is magnified to observe the structure and color from the epidermis to the papillary layer (6, 7). This technique is extensively used in the diagnosis of skin-related tumors and hair-related and inflammatory skin diseases (8–10). However, reports on the application of dermoscopy in CLC diagnosis are scarce. Yellowish lacunae surrounded by a pale septum and pale red to bluish lacunae in CLC can be observed *via* dermoscopy (11). Some atypical lesions might be diagnosed as CLC by using adjunctive measures, such as dermoscopy and histology (12). In hypopyon-like types of CLC patterns, CLC was found to contain blood components. CD31 is a highly glycosylated Ig-like membrane receptor. It is the most abundant membrane glycoprotein constitutively expressed on the vascular endothelium (13). Therefore, we used immunohistochemistry to detect CD31 expression. This study aimed to examine dermoscopic CLC manifestations retrospectively, create a complement to existing CLC dermoscopic features, and understand why some CLC subtypes are manifested with blood components.

## Methods

After institutional review board approval, we viewed the pathological databases, outpatient records, and dermoscopic systems in Xiangya Hospital, Central South University and Hunan Province Children's Hospital from July 2017 to July 2019. Any patients with detailed demographic information (age, gender, and location), biopsy specimens, and dermoscopic and clinical data were included in this study. As a result, 584 patients with CLC were chosen. Next, patients were carefully excluded if any of their demographic information, specimens, and dermoscopic and clinical data were not available. Finally, only 37 patients were included in this study.

Nevertheless, other patients with CLC who had detailed demographic information (age, gender, and location), biopsy specimens, and clinical data were included in the subsequent immunohistochemical staining study. A total of 62 specimens were immune-stained with CD31 (a blood vessels marker) and D2-40 (a lymphatic vessels marker). All the patients provided informed consent. The use of the slides of these patients was approved by the Medical Ethical Committee of the hospitals and was conducted in accordance with the Declaration of Helsinki guidelines.

The final diagnosis of CLC was made by two professional dermato-pathologists (Professor Mingliang Chen and Dr. Xiaoyan Huang). At the time of the histologic and immunohistochemistry analyses, the dermato-pathologists were blinded to the clinical data. Thereafter, the electronic medical records were reviewed by Professor Juan Su and Dr. Lixia Lu for available clinical data on age, gender, clinical morphology, anatomic distribution, symptoms, and duration of clinical findings. The clinical images and dermoscopic features were assessed by two professional dermatologists (Dr. Lixia Lu and Dr. Siyun Yan). Medicam 800/1,000 (FotoFinder, Bad Birnbach, Germany) was used as the dermatoscopic instrument.

SPSS statistics 23.0 was used for the statistical analysis. A chi-square test was used to compare the differences among various groups. *p* < 0.05 was considered statistically significant.

## Results

**Figure 1:**
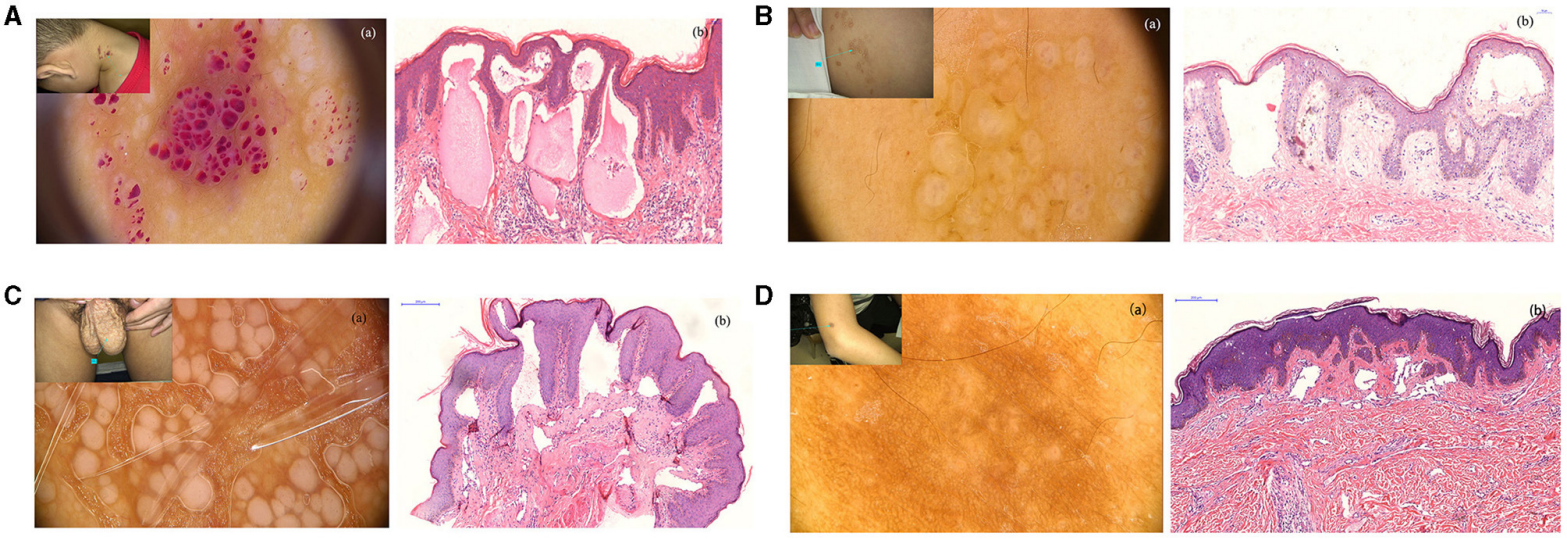
**(A)** Clinical manifestations of patients with cutaneous lymphangioma circumscriptum (CLC), a cluster of vesicular dark reddish/complexion on the neck for 3 years; (a) reddish, white-yellowish lacuna of different sizes, some filled with blood as hypopyon-like or crescent shapes (non-polarized, 20×); (b) dilated lymphatic vessels with various sizes, containing lymph, erythrocytes, and inflammatory cells. (H&E stain; original magnification, 200×) (300 dpi × 300 dpi). **(B)** Clinical manifestations of patients with CLC, lacunae on the back of the left shoulder for 2 years. The patient had a history of CLC and treatment with surgery. (a) Dermatoscopic features of patients with CLC: blister resembling frog spawn or translucent vesicles (non-polarized, 20×). (b) A representative histological analysis of patients with CLC shows the lumen of different sizes and lymphangiectasis. (H&E stain; original magnification, 200×) (300 dpi × 300 dpi). **(C)** Clinical manifestations of patients with CLC, papules on the scrotum for several years, liquid intermittent efflux. (a) White unstructured areas of varying sizes, independent of each other (non-polarized, 40×). (b) A representative histological analysis of patients with CLC shown in dilated lymphatic vessels and immunohistochemical staining, showing positive D2-40 staining (H&E stain; original magnification, 100×) (300 dpi × 300 dpi). **(D)** Clinical manifestations of patients with CLC (A), brown patch enlarging gradually on the right arm. (a) White unstructured areas in the center, brown reticular pigmentation in the periphery (non-polarized, 70×). (b) A representative histological analysis of patients with CLC shown in lymphangiectasis, accompanied with fibroplasia (H&E stain; original magnification, 100×) (300 dpi × 300 dpi).

The 37 patients who were diagnosed with CLC revealed the four types of dermoscopy features. Type 1 presented with clusters of dark reddish/complexion vesicles. Several lacunae were observed to be clustered like rice (Figure 1Aa, upper left). Dermoscopy revealed multiple reddish, white-yellowish lacunae of different sizes, some of which were filled with blood components with hypopyon-like or crescent shapes. Several lacunae contained variable amounts of blood and blood sediments, resulting in an appearance similar to that of “half-and-half” blisters (Figure 1Aa). H&E staining revealed dilated lymphatic vessels that contain lymph and inflammatory cells (Figure 1Ab).

Type 2 presented with complexion papules/masses. The lesions were translucent and manifested as multiple blisters that partially fused into a patch and were partially independent (Figure 1Ba, upper left). Blisters resembling frog spawn or translucent vesicles were observed without blood inclusions; dermoscopic examination showed representative changes in CLC (Figure 1Ba). Histological analysis also verified the dermoscopic diagnosis, whereas H&E staining revealed dilated lymphatic vessels with lymphatic fluids (Figure 1Bb).

Type 3 showed white papules of varying sizes, with intermittent liquid efflux on the scrotum (Figure 1C, upper left). The independently distributed milky/whitish lacunae of different sizes and the punctate and dilated blood vessels around the papules were observed *via* dermoscopy (Figure 1Ca). Histological analysis revealed the dilated lymphatic vessels that were positive for D2-40 (Figure 1Cb).

The last type presented with a black/brown patch that had a clear boundary and a slightly hard texture on the right upper arm (Figure 1Da, upper left). This patch initially occurred as a black papule and gradually changed to a black and brown patch as it enlarged. Dermoscopic observations showed white-like circular unstructured areas distributed in the center and brown reticulate pigmentations in the periphery (Figure 1Da). Dermatofibroma was considered upon dermoscopic examination. However, dilated lymphatic vessels and fibroplasia were observed by H&E staining (Figure 1Db). Hence, the final diagnosis was CLC.

The detailed clinical manifestations are summarized in Table 1. The type of blister fills with a hypopyon-like/moon shape and red zone (46.0%, 17/37) and blisters resembling frog spawns (29.7%, 11/37) were the most common dermoscopic manifestations, accounting for 75.7% of the cases. The lesions were mainly distributed in the trunk (40.5%, 15/37) and limbs (29.7%, 11/37). Moreover, differential clinical diagnoses included angiokeratoma, hemangioma, verruca vulgaris, venous malformation, melanoma, skin mucinosis, and nevus. The former three diagnoses were the most common differential diagnoses.

**Table 1:** The clinical manifestations, dermatoscopic findings, and histological features of patients with cutaneous lymphangioma circumscriptum (CLC).

**Dermatoscopic** **features**	**Age,** **y Mean ± SD(range)**	**Frequency** ***n* (%)**	**Gender (M:F)**	**Location**	**Clinical features**	**Primary diagnosis/** **differential diagnosis**	**Histology**
Hypopyon-like/crescent shape	20.4 ± 9.6 (5–36)	17 (46.0%)	1:2	Limbs (8)Trunk (5)Head and Neck (4)	Reddish and complexion papule/ plaque/mass/ Vesicular/lacunae	Angiokeratoma, Hemangioma, CLC, Verruca plana, Venous malformation, Melanoma	Dilated lymphatics, some containing lymph, erythrocytes and inflammatory cells
Blister resembling frog spawn or translucent vesicle	17.8 ± 8.6 (4–25)	11 (29.7%)	1:2.7	Limbs (3)Trunk (5)Head and Neck (3)	Complexion/yellowish/ Translucentpapule/ plaque/mass/ Vesicular/lacunae	Angiokeratoma, CLC, Hemangioma, Verruca plana, herpes zoster	Lymphangiectasia of different size
Milky/whitish lacunae	20 ± 4.9 (17–30)	5 (13.5%)	1:1.5	Scrotum (2)Trunk (3)	Papules, intermittent liquid efflux	Skin mucinosis	Dilated lymphatic vessels and D2-40 staining was positive
Brown reticulate pigmentation	24 ± 3.7 (20–36)	4 (10.8%)	1:1	Limbs (2)Trunk (2)	Brown patch, gradually enlarge	Dermatofibroma, Nevus	lymphangiectasia, accompanying with fibroplasia

The reasons that lead to the clinical manifestations of lymphangioma as dermoscopic type 1, hypopyon-like or crescent shape, were investigated. The blood component as a contributor to lymphangioma subtypes was investigated *via* immunohistochemistry to detect CD31. Combining CD31 expression and clinical manifestations, CD31 was detected in the inner flattened endothelium of the CLC lesions and was only obvious in cystic-dilated spaces, containing sufficient blood components or lymph.

**Figure 2:**
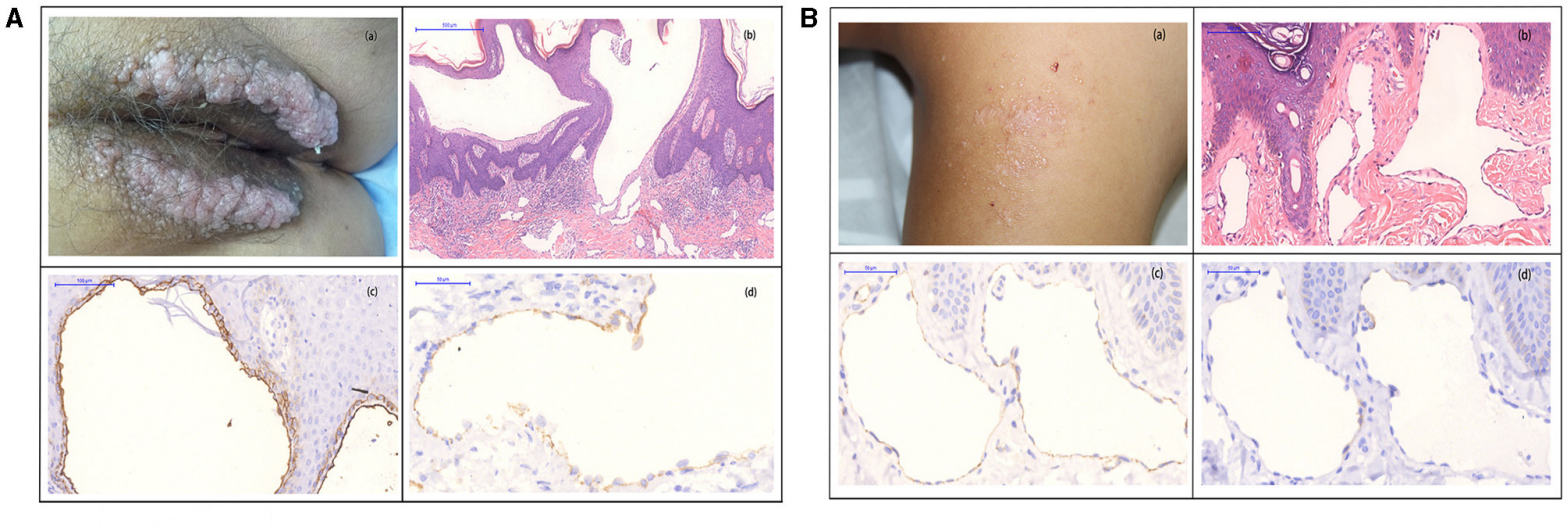
**(A)** (a) Biopsy specimens were taken from transparent blister-like beads on a string from the vulva. (b) H&E staining showed cystic-dilated spaces, containing numerous erythrocytes and leukocytes in the papillary and upper reticular dermis (original magnification, 100×). (c) D2-40 staining showed two layers of the inner (red arrow) and outer (black arrow)-flattened endothelial (original magnification, 200×). (d) Immunohistochemistry showed CD31 expression in the inner-flattened endothelium (original magnification, 400×) (300 dpi × 300 dpi). **(B)** (a) Biopsy specimens were taken from the complexion papule in the dorsum, and the papule has a little lymph. (b) H&E staining showed evidently extended lymphatic vessels in the papillary and upper dermis (original magnification, 200 ×). (c) High magnification of D2-40 staining showed a single endothelial layer (red arrow), the absence of two endothelial layers of the inner- and outer-flattened endothelium (original magnification, 400×). (d) Immunohistochemical staining for CD31 showed negative expression in the flattened endothelium (original magnification, 400×) (300 dpi × 300 dpi).

A female patient presented with transparent blister-like beads on a string in the vulva for 3 years (Figure 2Aa), accompanied by pain and transparent secretion sometimes. H&E staining showed extended lymphatic vessels in the papillary and upper dermis and the lymph run-off (Figure 2Ab). The immunohistochemistry for D2-40 revealed two endothelial layers (inner and outer endothelial), separated by a fluid phase, containing abundant erythrocytes and lymph. The inner endothelium is flattened, whereas the outer layer is rounded (Figure 2Ac). The two endothelial layers were absent in the space, containing few or no blood and lymph. CD31 staining showed expression in the inner-flattened endothelium (Figure 2Ad).

Another male patient presented to us with a small and flattened complexion papule in the dorsum for 5 years (Figure 2Ba). A laser was used, but it was recurrent after 3 months of treatment. H&E staining showed evidently extended lymphatic vessels in the papillary and upper dermis (Figure 2Bb). D2-40 staining presented a single endothelial layer, but no two distinctive layers (Figure 2Bc). CD31 staining showed no expression in the flattened endothelium (Figure 2Bd).

Associations among the clinical manifestation and clinic-pathological characteristics of lymphangioma are summarized in Table 2. The median age was 31 years old, and males accounted for the majority of the patients (67.7%, 42/62). Lesions were mainly distributed in the trunk and extremities (85.4%, 53/62). The patients were then divided into two groups according to their clinical manifestations, namely, with and without abundant blood components or lymph. The variables (age, gender, and location) of the patients showed no statistical significance. However, CD31 staining was positive, and the two endothelial layers were obvious in the spaces containing erythrocytes and lymph. This phenomenon was not observed in lymphangioma characterized by the absence of blood and lymph.

**Table 2:** Associations between the clinical manifestation and clinicopathologic characteristics of lymphangioma cases.

**Variables**	**Clinical manifestation**			*P*-value^*^
	**With enough** **erythrocyte/lymph (*N* = 34)**	**Without enough** **erythrocyte/lymph (*N* = 28)**	**Total**	
**Age, years (** ***N*** **, %)**				0.37
<31	22 (35.5%)	15 (24.2%)	37 (59.7%)	
≥31	12 (19.4%)	13 (21.0%)	25 (40.3%)	
**Gender (** ***N*** **, %)**				0.28
Male	25 (40.3%)	17 (27.4%)	42 (67.7%)	
Female	9 (14.5%)	11 (17.7%)	20 (32.3%)	
**Location (** ***N*** **, %)**				0.12
Head and neck	3 (4.8%)	6 (9.7%)	9 (14.5%)	
Trunk (including vulva)	19 (30.6%)	14 (22.6%)	33 (53.2%)	
Extremities	12 (16.1%)	8 (12.9%)	20 (32.3%)	
**CD31 staining (N, %)**				**1.120E-07**
Positive	29 (46.8%)	4 (6.5%)	33 (53.2%)	
Negative	5 (8.1%)	24 (38.7%)	29 (46.8%)	
**Endothelia layer (** ***N*** **, %)**				**3.6945E-09**
Monolayer	28 (45.2%)	2 (3.2%)	30 (87.1%)	
Multilayer	6 (9.7%)	26 (41.9%)	32 (12.9%)	

*Analysis by χ^2^ test or Fisher's exact-test; For the 62 lymphangioma cases, the median age of the whole group of patients was 31 years (range, 4–78 years)*.

* *significant association, if present. The bold values means significant association, if present*.

## Discussion

This work systematically reviewed the available evidence of the dermoscopic structures among lymphangiomas and investigated why some lymphangiomas are marked by blood components. We speculated that these characteristics can be used to differentiate CLC from other diseases, including angiokeratomas, venous malformations, melanomas, hemangiomas, verruca plana, and herpes zoster. A lymphangioma is a benign tumor that occurs in the lymphatic system. It is regarded as a congenital malformation of the lymphatic vessel or lymphatic vessel hyperplasia. Clinically and pathologically, a lymphangioma is divided into three categories: simple lymphangioma, cavernous lymphangioma, and cystic lymphangioma associated with lymphatic malformation (14). A lymphangioma is characterized by lesions involving lymphatic and blood vessels. The histopathological features of CLC are presented as highly dilated lymphatic vessels lined with squamous epithelial cells, which are rich with lymphatic liquid and lymphocytes and sometimes mixed with blood (15).

Dermoscopy is a non-invasive, *in vivo* imaging technique that allows for the visualization of subsurface skin structures. According to the literature, CLC displays two distinct dermoscopical patterns: the yellow-pale septa pattern in the absence of erythrocytes and the yellow to red/pink/bluish lacunae pattern due to the presence of blood (15, 16). Our study also verified that these two patterns had the highest proportion in the patients, accounting for 75.5% of the total cases. Aside from the common dermoscopic patterns, two previously undescribed types of CLC, namely, milky/whitish lacunae and brown reticulate pigmentations, were also observed. The milky/whitish lacunae pattern must be differentiated from skin mucinosis, whereas the brown reticulate pattern should be differentiated from dermatofibroma and pigmented nevus. The deepest skin structure observed by dermoscopy was the papilla layer; so, when the dilated lymphatic vessels are located under the dermal papilla, the dermoscopy cannot observe it well. Our study presents a summary and a supplement to existing dermoscopic manifestations of lymphangioma and provides assistance in the diagnosis of lymphoma and selection of the appropriate treatments. As we all know, the recurrence rate is high in CLC (17). A retrospective study showed that a recurrent rate of 29% was found in 196 lesions of 186 patients during 3 years. Approximately 60% of patients with relapse recurred within 1 year and 80% recurred within 3 years (18). Therefore, follow-up is necessary.

Among the 584 patients with lymphangioma, only 37 patients were examined *via* dermoscopy, suggesting that the use of dermoscopy needs to be improved. Dermoscopy can distinguish CLC from hemangiomas, melanomas, and other phenotypically similar skin lesions. There are no departments of pathology in primary hospitals and township health centers. Hence, dermoscopy can be a useful tool in the diagnosis of CLC that may, otherwise, be missed in hospitals without pathology departments. However, dermoscopy can only reflect the pathological mapping of superficial skin lesions. Thus, dermoscopy can sometimes easily lead to misdiagnosis. Clinical examination should be combined with various methods, and a biopsy should be performed as necessary to make a correct diagnosis.

The reason CLC contains blood components was explored *via* immunohistochemistry to detect CD31 expression in CLC. Bhawan et al. reported CD31 expression in endothelial cells in lymphangiomas (19). However, the relationship between CD31 expression and the clinical manifestations of CLC remains uncertain. Our results showed that the two endothelial layers were only obvious in cystic-dilated spaces, containing abundant erythrocytes or lymph. Oiso et al. reported that a lymphangioma with reddish vesicles contains two flattened endothelial layers, but the lumen of yellowish vesicles lacks such manifestations (12). Our results were consistent with that observation. The double layer of endothelial cells in the lumen with hypopyon-like or crescent-shaped CLC was also found in cystic-dilated spaces filled with lymph. Our results may shed light on the pathogenesis of frequent blood leakage inside dilated spaces in CLC.

## Data Availability Statement

The original contributions presented in the study are included in the article/supplementary material, further inquiries can be directed to the corresponding authors.

## Ethics Statement

The patients in this manuscript have given written informed consent to publication of their case details and the use of patients' biopsy slides was approved by the Medical Ethical Committee of Xiangya hospital, Central South University and was conducted per the Declaration of Helsinki guidelines.

## Author Contributions

LL and SY: writing-review and editing, preparation, creation and/or presentation of the published work by those from the original research group, specifically critical review, commentary, or revision - including pre- or post-publication stages, investigation, conducting a research and investigation process, specifically performing the experiments, or data/evidence collection, and formal analysis, application of statistical, mathematical, computational, or other formal techniques to analyze or synthesize study data. LL, JS, SY, and XH: writing-original draft preparation, creation and/or presentation of the published work, and specifically writing the initial draft (including substantive translation). JS and XH: supervision, oversight and leadership responsibility for the research activity planning and execution, and including mentorship external to the core team. LL, SY, and MC: data curation, management activities to annotate (produce metadata), scrub data, and maintain research data (including software code, where it is necessary for interpreting the data itself) for initial use and later reuse. JS and XH: conceptualization, ideas and formulation or evolution of overarching research goals and aims. All authors contributed to the article and approved the submitted version.

## Funding

This study was funded by grants from the National Natural Science Foundation of China (Grant No. 81974478 to JS) and the Hunan Provincial Natural Science Fund (Grant No. 2019JJ40498 to MC).

## Conflict of Interest

The authors declare that the research was conducted in the absence of any commercial or financial relationships that could be construed as a potential conflict of interest.

## Publisher's Note

All claims expressed in this article are solely those of the authors and do not necessarily represent those of their affiliated organizations, or those of the publisher, the editors and the reviewers. Any product that may be evaluated in this article, or claim that may be made by its manufacturer, is not guaranteed or endorsed by the publisher.
